# Magnetic resonance imaging for treatment response evaluation and prognostication of hepatocellular carcinoma after thermal ablation

**DOI:** 10.1186/s13244-023-01440-7

**Published:** 2023-05-16

**Authors:** Yun Zhang, Hong Wei, Bin Song

**Affiliations:** 1 Department of Radiology, West China Hospital, Sichuan University, No. 37, Guoxue Alley, Chengdu, 610041 Sichuan China; 2 Department of Radiology, Sanya People’s Hospital, Sanya, Hainan China

**Keywords:** Carcinoma, Hepatocellular, Ablation, Magnetic resonance imaging, Treatment response, Prognosis

## Abstract

**Abstract:**

Hepatocellular carcinoma (HCC) accounts for the vast majority of primary liver cancer and constitutes a major global health challenge. Tumor ablation with either radiofrequency ablation (RFA) or microwave ablation (MWA) is recommended as a curative-intent treatment for early-stage HCC. Given the widespread use of thermal ablation in routine clinical practice, accurate evaluation of treatment response and patient outcomes has become crucial in optimizing individualized management strategies. Noninvasive imaging occupies the central role in the routine management of patients with HCC. Magnetic resonance imaging (MRI) could provide full wealth of information with respect to tumor morphology, hemodynamics, function and metabolism. With accumulation of liver MR imaging data, radiomics analysis has been increasingly applied to capture tumor heterogeneity and provide prognostication by extracting high-throughput quantitative imaging features from digital medical images. Emerging evidence suggests the potential role of several qualitative, quantitative and radiomic MRI features in prediction of treatment response and patient prognosis after ablation of HCC. Understanding the advancements of MRI in the evaluation of ablated HCCs may facilitate optimal patient care and improved outcomes. This review provides an overview of the emerging role of MRI in treatment response evaluation and prognostication of HCC patients undergoing ablation.

**Clinical relevance statement:**

MRI-based parameters can help predict treatment response and patient prognosis after HCC ablation and thus guide treatment planning.

**Key points:**

1. ECA-MRI provides morphological and hemodynamic assessment of ablated HCC.
2. EOB-MRI provides more information for tumor response prediction after ablation.
3. DWI improve the characterization of HCC and optimize treatment decision.
4. Radiomics analysis enables characterization of tumor heterogeneity guidance of clinical decision-making.
5. Further studies with multiple radiologists and sufficient follow-up period are needed.

**Graphical abstract:**

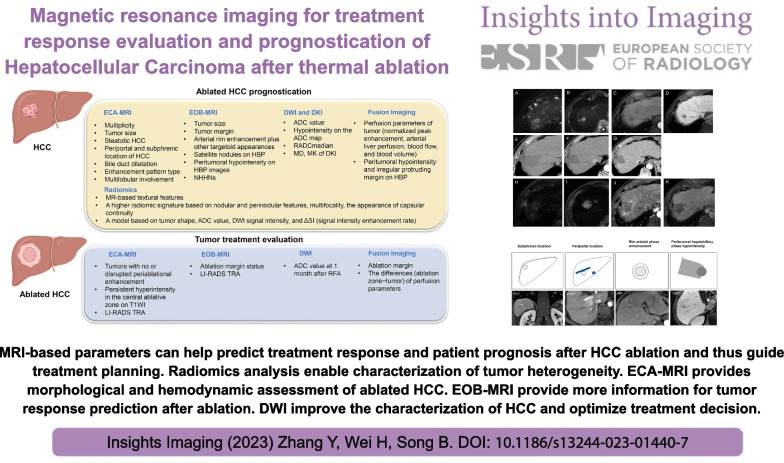

## Background

Liver cancer is the second most lethal malignancy globally and its incidence is on the rise, with more than 1 million worldwide new cases by 2025 [1, 2]. Hepatocellular carcinoma (HCC) accounts for the vast majority of primary liver cancer and constitutes a major global health challenge [1]. Tumor ablation with either radiofrequency ablation (RFA) or microwave ablation (MWA) is recommended as a curative-intent treatment for early-stage HCC, and frequently used to downsize or control tumor burden prior to liver transplantation [2, 3]. Particularly, ablation could be given priority over hepatectomy for patients with HCC ≤ 3 cm owing to the merits of comparable survival benefits, less invasiveness and cost-effectiveness [4]. Given the increased application of thermal ablation in routine clinical practice, accurate evaluation of treatment response and patient outcomes has become crucial in optimizing personalized management strategies.

**Fig. 1:**
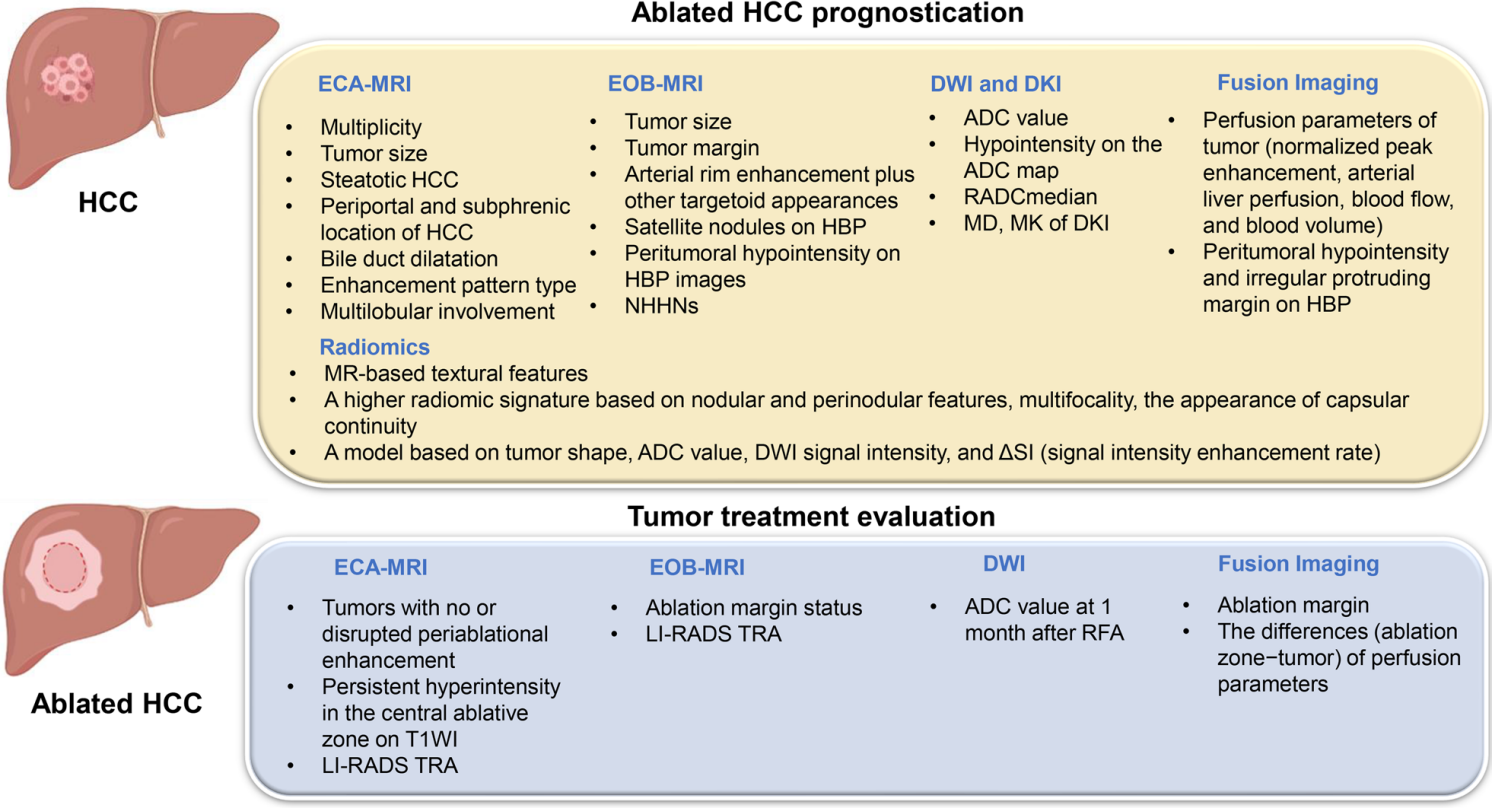
Published magnetic resonance imaging features for treatment response evaluation and prognostication of hepatocellular carcinoma after thermal ablation. RADC_median_ was defined as the ratio of ADC_median_ to the mean ADC of the non-lesion area

**Fig. 2:**
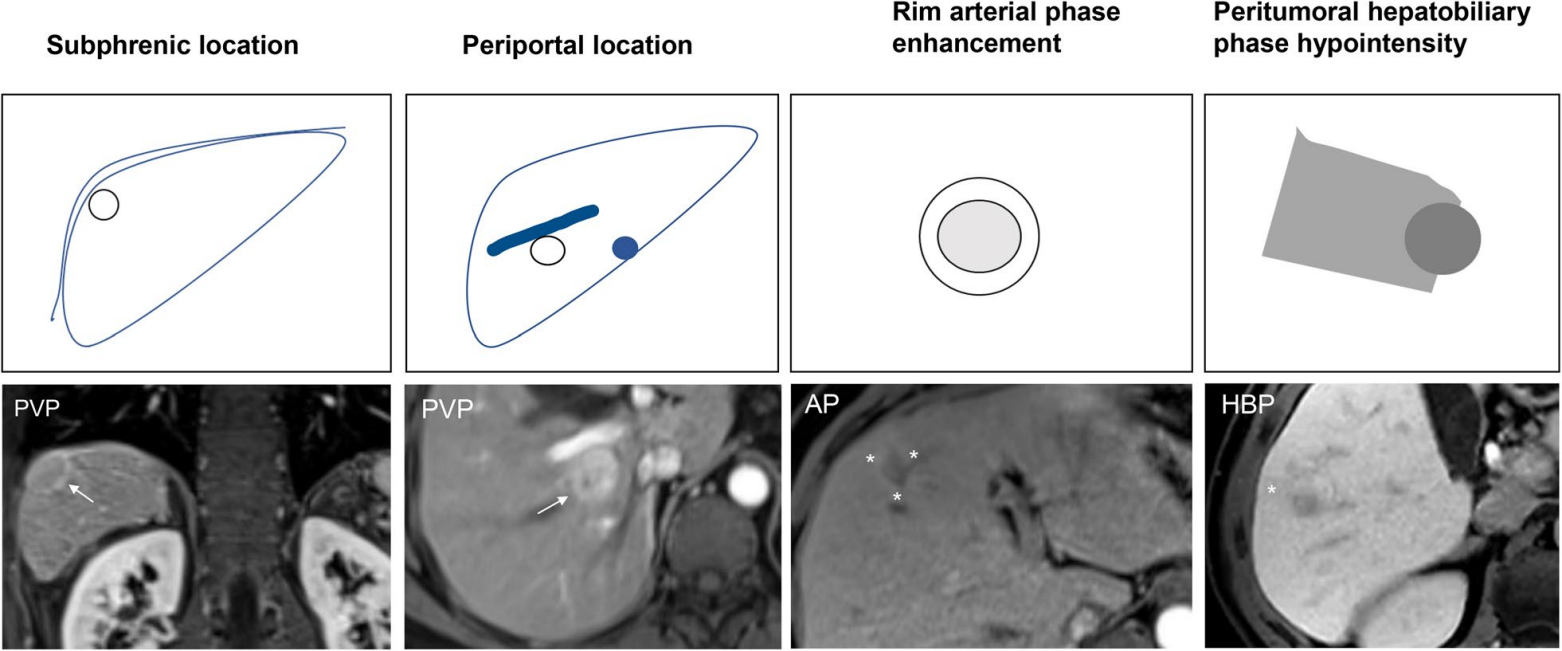
Graphical illustration of some qualitative MR features of HCC associated with poor outcomes after ablation treatment. PVP, portal venous phase. AP, arterial phase. HBP, hepatobiliary phase

Noninvasive imaging plays a critical role in the therapeutic response assessment and risk stratification of HCC, and magnetic resonance imaging (MRI) exhibits a particularly promising prospect. Conventional extracellular contrast agent-enhanced MRI (ECA-MRI) has been widely used for qualitative evaluation of tumor morphology and hemodynamics. With tremendous progress in MRI techniques, and the introduction of hepatospecific agents such as gadoxetate disodium (Eovist/Primovist; Bayer HealthCare, Berlin, Germany)-enhanced MRI (EOB-MRI), the diagnosis and characterization of HCC has improved significantly [5–7]. On the basis of this, EOB-MRI combined with diffusion-weighted imaging (DWI) can further improve the capabilities of HCC characterization and enabled accurate guidance of ablation treatment plan by providing images with high tumor-to-liver contrast and good depiction of intrahepatic vascular and biliary structures [8–10]. With accumulation of liver MR imaging data, radiomics analysis has recently emerged as a promising strategy that enables characterization of tumor heterogeneity and guidance of clinical decision-making by extracting high-throughput quantitative imaging features from digital medical images, including signal intensity, histogram-based features, and textural feature [11–13]. Encouraging studies have been published on the potential utility of novel MRI characteristics, including qualitative features, quantitative parameters and radiomic signatures, for noninvasively estimating therapeutic efficacy and providing prognostication in HCC patients treated with ablation (Figs. 1 and 2, Tables 1 and 2). In this context, understanding the usefulness of MRI for evaluation of ablated HCCs may contribute to the optimal clinical decision-making and improved patient outcomes. The purpose of this review is to overview of the emerging role of MRI in treatment response evaluation and prognostication of HCC patients undergoing ablation therapy.

**Table 1:** Summary of published studies focusing on magnetic resonance imaging for treatment response evaluation and prognostication of hepatocellular carcinoma after thermal ablation

Reference	Publication year	Study design	Area	No. of patients	No. and type of lesions	Imaging modality	Study endpoints	Ablation modality
Sheng et al. [14]	2015	Retrospective, single-center	China	35	40 HCCs	ECA-MRI	Prediction of IDR	RFA
Hermida et al. [15]	2021	Retrospective, single-center	France	238	412 HCCs	CEMRI; CECT	Evaluation of LTP, Time-to-LTP, IDR, Time-to-IDR, RFS and OS	RFA; MWA
Hermida et al. [16]	2020	Retrospective, single-center	France	235	419 HCCs	ECA-MRI	Evaluation of TTR, RFS and OS	RFA; MWA
Chaudhry et al. [18]	2020	Retrospective, single-center	USA	36	53 HCCs	ECA-MRI	Evaluation of the performance of LI-RADS version 2018 TRA	RFA; MWA
Bae et al. [19]	2021	Retrospective, single-center	Korea	183	HCC^†^	EOB-MRI	Prediction of DFS and OS	RFA
Cha et al. [20]	2021	Retrospective, single-center	Korea	349	349 HCCs	EOB-MRI	Evaluation of early recurrence	RFA
Kang et al. [21]	2016	Retrospective, single-center	Korea	211	211 HCCs	EOB-MRI	Prediction of LTP	RFA
Cools et al. [22]	2020	Retrospective, single-center	USA	45	81 HCCs	EOB-MRI	Validation of LI-RADS version 2018 TRA	RFA; MWA
Lee et al. [26]	2015	Retrospective, single-center	Korea	139	178 HCCs	EOB-MRI	Prediction of Recurrence	RFA
Toyoda et al. [27]	2015	Prospective, single-center	Japan	138	HCC^†^	EOB-MRI	Prediction of Recurrence	RFA
Iwamoto et al. [28]	2017	Retrospective, single-center	Japan	91	115 HCCs	EOB-MRI	Prediction of IDR	RFA
Inoue et al. [29]	2017	Retrospective, single-center	Japan	132	HCC^†^	EOB-MRI	Prediction of IDR	RFA
Lee et al. [34]	2019	Retrospective, single-center	Korea	345	345 HCCs	EOB-MRI	Evaluation of RFS	RFA
Koda et al. [39]	2015	Prospective, single-center	Japan	95	124 HCCs	EOB-MRI	Evaluation of LTP	RFA
Takeyama et al. [40]	2019	Retrospective, single-center	Japan	29	59 HCCs	EOB-MRI	Prediction of LTP	RFA
Lee et al. [41]	2020	Retrospective, single-center	Korea	467	467 HCCs	CEMRI; CECT	Evaluation of LTP and OS	RFA
Kawamura et al. [45]	2019	Retrospective, single-center	Japan	488	488 HCCs	ECA-MRI; CECT	Prediction of intrasubsegmental recurrence	RFA
Kondo et al. [46]	2011	Prospective, single-center	Japan	589	HCC^†^	ECA-MRI; CECT	Evaluation of recurrence and survival	RFA
Kim et al. [47]	2017	Prospective, single-center	Korea	33	42 HCCs	ECA-MRI; CECT	Evaluation of LTP	RFA
Mori et al. [49]	2015	NA	Japan	136	168 HCCs	DWI	Evaluation of recurrence and survival	RFA
Ma et al. [50]	2019	Retrospective, single-center	China	64	HCC^†^	DWI	Prediction of tumor progression	RFA
Barat et al. [51]	2017	Retrospective, dual-center	France	59	38 HCCs; 27 metastases	DWI	Evaluation of local tumor recurrence	RFA
Hu et al. [52]	2020	Retrospective, single-center	China	105	HCC^†^	ECA-MRI	Prediction of LTP	RFA
Yuan et al. [55]	2019	Retrospective, single-center	China	107	107 HCCs	DKI; DWI	Prediction of recurrence	RFA
Kobe et al. [57]	2021	Retrospective, single-center	Switzerland	39	43 HCCs	ECA-MRI; Perfusion CT	Evaluation of local tumor recurrence	RFA
Yoon et al. [58]	2018	Prospective, single-center	Korea	68	88 HCCs	EOB-MRI; CECT	Evaluation of LTP	RFA
Wang et al. [59]	2020	Retrospective, single-center	China	115	115 HCCs	EOB-MRI; CEUS	Evaluation of recurrence	RFA
Horvat et al. [60]	2021	Retrospective, single-center	Brazil	34	51 LR-4/5 nodules	ECA-MRI	Prediction of sustained complete response	RFA
Petukhova-Greenstein et al. [61]	2022	Retrospective	Germany	65	85 HCCs	ECA-MRI	Prediction of PFS	RFA
Wen et al. [62]	2021	Retrospective, single-center	China	111	HCC^†^	GD-MRI	Prediction of early recurrence	RFA
Lv et al. [63]	2021	Retrospective, single-center	China	58	HCC^†^	ECA-MRI	Prediction of aggressive intrasegmental recurrence	RFA

*CECT* contrast-enhanced computed tomography; *CEMRI* contrast-enhanced magnetic resonance imaging; *CEUS* contrast-enhanced Ultrasound; *CT* computed tomography; *DFS* disease-free survival; *DKI* diffusion kurtosis imaging; *DWI* diffusion-weighted imaging; *ECA-MRI* extracellular contrast agent-enhanced magnetic resonance imaging; *EOB-MRI* gadoxetate disodium-enhanced magnetic resonance imaging; *GD-MRI* gadobenate dimeglumine-enhanced magnetic resonance imaging; *HCC* hepatocellular carcinoma; *IDR* intrahepatic distant recurrence; *LI-RADS/LR* Liver Imaging Reporting and Data System; *LTP* local tumor progression; *MRI* magnetic resonance imaging; *MWA* microwave ablation; *NA* not available; *OS* overall survival; *PFS* progression-free survival; *RFA* radiofrequency ablation; *RFS* recurrence-free survival; *SPIO* superparamagnetic iron oxide; *TRA* treatment response algorithm; *TTR* time to recurrence

^†^The number of HCC is unavailable

**Table 2:** Summary of key results in published studies focusing on magnetic resonance imaging for treatment response evaluation and prognostication of hepatocellular carcinoma after thermal ablation

Reference	Imaging finding	Clinical parameter	Primary outcome
Sheng et al. [14]	Multiplicity, tumors with no or disrupted periablational enhancement, and persistent hyperintensity in the central ablative zone on T1WI	Serum albumin < 3.5 g/dL	IDR
Hermida et al. [15]	Tumor size Tumor size Multiplicity Multiplicity and steatotic HCC	Ultrasound guidance AFP > 100 ng/mL Treatment naivety and AFP > 100 ng/mL ASA score > 2 and AFP	LTP and time-to-LTP IDR Time-to-IDR and RFS OS
Hermida et al. [16]	Steatotic HCC	AFP	OS
Chaudhry et al. [18]	LI-RADS TRA, and arterial phase hyperenhancement		Histopathological tumor necrosis
Bae et al. [19]	Satellite nodules on HBP images Satellite nodules and peritumoral hypointensity on HBP images	Serum albumin Serum albumin and PT-INR	DFS OS
Cha et al. [20]	Arterial rim enhancement plus other targetoid appearances		LTP, IDR, and EM within 2 years
Kang et al. [21]	Tumor size, tumor margin and HBP peritumoral hypointensity		LTP
Cools et al. [22]	LI-RADS TRA		Residual viable tumors at histopathology
Lee et al. [26]	NHHNs	Prothrombin activity and LTP Child–Pugh class and EM	RFS OS
Toyoda et al. [27]	NHHNs		Recurrence
Iwamoto et al. [28]	NHHNs	Child–Pugh class (B)	IDR and new intrahepatic recurrence
Inoue et al. [29]	NHHNs	Child–Pugh class (B)	IDR
Lee et al. [34]	NHHNs -Presence of NHHNs -Absence of NHHNs		RFS after resection and RFA -Similar 5-year RFS after resection and RFA -Better 5-year RFS after resection versus RFA
Koda et al. [39]	Ablation margin grading, and tumor size^§^		LTP
Takeyama et al. [40]	Ablation margin status		LTP
Lee et al. [41]	Periportal and subphrenic location of HCC and tumor size	HCV infection, Child–Pugh class B, platelet count, LTP, IDR, AIR, and EM	LTP OS
Kawamura et al. [45]	Enhancement pattern type	Treatment procedure (touch ablation), and AFP ≥ 30 μg/L	Intrasubsegmental recurrence
Kondo et al. [46]	Bile duct dilatation affecting two or more subsegments, and tumor number Bile duct dilatation affecting two or more subsegments, and tumor number	HCV infection, Child–Pugh class (B or C), AFP > 100 ng/mL, and DCP ≥ 100 mAu/mL HCV infection, Child–Pugh class (B or C), and DCP ≥ 100 mAu/mL	Recurrence Death
Kim et al. [47]	Ablation margin status		LTP
Mori et al. [49]	Hypointensity on the ADC map and tumor number Hypointensity on the ADC map	Etiology (HCV) Type IV collagen 7S and AFP	Recurrence Survival
Ma et al. [50]	RADC_median_^†^		Tumor progression
Barat et al. [51]	ADC value at 1 month after RFA		Recurrence
Hu et al. [52]	ADC value and rim enhancement		LTP
Yuan et al. [55]	ADC, MD, MK		Recurrence
Kobe et al. [57]	The differences (ablation zone-tumor) of the perfusion parameters		LTR
Yoon et al. [58]	Ablation margin assessed by registration software		LTP
Wang et al. [59]	Peritumoral hypointensity and irregular protruding margin on HBP		Recurrence
Horvat et al. [60]	Textural features		Treatment response
Petukhova-Greenstein et al. [61]	A higher radiomic signature based on nodular and perinodular features, multifocality, the appearance of capsular continuity		PFS
Wen et al. [62]	Radiomics signature	Platelet count	Early recurrence
Lv et al. [63]	Radiomics signature, tumor shape, ADC value, DWI signal intensity, and ΔSI (signal intensity enhancement rate)		AIR

*ADC* apparent diffusion coefficient; *AFP* alpha fetoprotein; *AIR* aggressive intrasegmental recurrence; *ASA* American Society of Anesthesiologists; *DCP* des-g-carboxy prothrombin; *DWI* diffusion weighted imaging; *EM* extrahepatic metastasis; *HBP* hepatobiliary phase; *HCV* hepatitis C virus; *IDR* intrahepatic distant recurrence; *LTP* local tumor progression; *LTR* local tumor recurrence; *MD* mean diffusion; *MK* mean kurtosis; *NHHN* nonhypervascular; *HBP* hypointense nodule; *PT-INR* prothrombin time-international normalized ratio; *WI* weighted imaging

^§^Tumor size was an independent predictor of LTP, whereas ablative margin grading was not an independent predictor of LTP

^†^RADC_median_ was defined as the ratio of ADC_median_ to the mean ADC of the non-lesion area

### Extracellular contrast agent-enhanced MRI

#### Conventional imaging markers

**Fig. 3:**
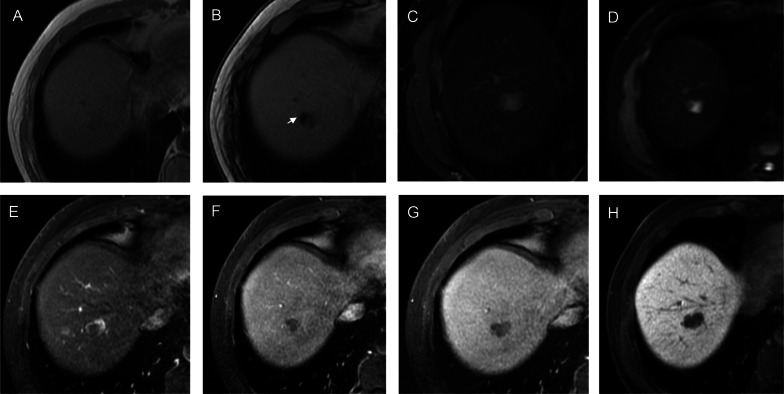
Gadobenate dimeglumine–enhanced axial MRI scans in a 57-year-old man with hepatitis B virus (A-H). A 1.7-cm steatotic mass is detected in segments VII. The mass shows T1 hypointensity (**A**), signal intensity reduced on out-of-phase TIWI (arrow in **B**), moderate T2 hyperintensity (**C**), marked hyperintensity on DWI (b = 1000 s/mm^2^) (**D**), rim enhancement on arterial phase image (**E**), “wash-out” appearance on portal venous phase (**F**) and equilibrium phase (**G**), marked hepatobiliary phase hypointensity (**H**). The recurrence-free survival for this patient was 150 days

Conventional imaging plays an important role in assessing and predicting the efficacy of ablative therapies for HCC because the images are stable and easily accessible, as well as the ease with which the image features obtained can be validated. A few attempts have been made to explore the potential of pretreatment ECA-MRI in evaluating the prognosis of HCC patients receiving ablation. In a retrospective, single-center study of 35 patients, Sheng et al. [14] demonstrated that multiplicity, tumors with no or disrupted periablational enhancement, persistent hyperintensity in the central ablative zone on T1 weighted imaging and serum albumin < 3.5 g/dL, were independently associated with intrahepatic distant metastasis after RFA of HCC. Moreover, another two retrospective studies showed that small steatotic HCC, identified on pretreatment MRI, was associated with a less aggressive tumor phenotype and improved overall survival [15, 16] (Fig. 3).

#### Liver imaging reporting and data system treatment response algorithm

**Fig. 4:**
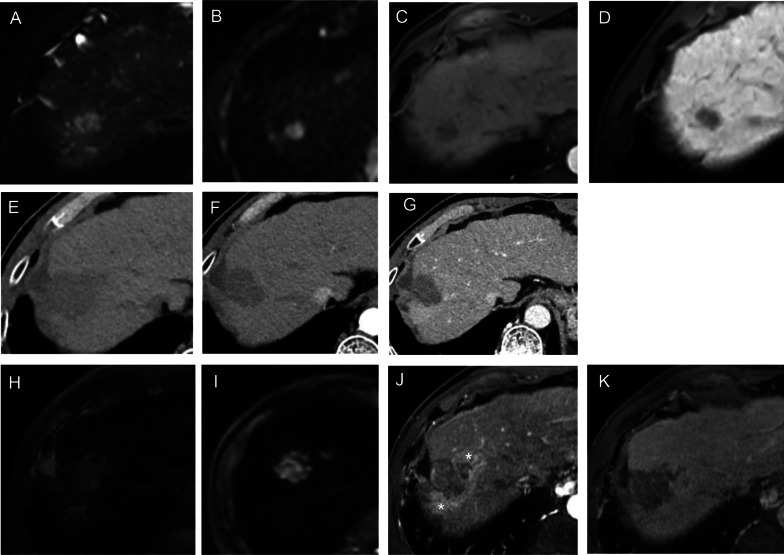
Images in a 56-year-old man with chronic hepatitis B virus infection. **A**–**D** Preoperative gadoxetate disodium–enhanced axial MRI scans show a 2.0-cm mass (NHHN) in segment VII, which shows moderate T2 hyperintensity (**A**), marked hyperintensity on DWI (b = 800 s/mm^2^) (**B**), without obvious enhancement on arterial phase image (**C**), marked hepatobiliary phase hypointensity (**D**). **E**–**G** Enhanced abdominal CT images of the patient 50 days after radiofrequency ablation treatment of tumor. A low-density ablation area was shown (**E**), and there was not any enhancement tissue in or along the margin of the treated lesion (**F**, **G**). Diagnosis was agreed upon by the 2 readers (LR-TR nonviable). **H**–**K** Gadoxetate disodium–enhanced axial MRI scans 67 days after tumor ablation, which shows T2 hyperintensity (**H**), marked hyperintensity on DWI (b = 800 s/mm^2^) (**I**), irregular thickened enhanced tissue area was found along the margin of the treated lesion (* in **J**), and marked hepatobiliary phase hypointensity (**K**). The recurrence-free survival for this patient was 60 days

Liver Imaging Reporting and Data System (LI-RADS) Treatment Response Algorithm (TRA) provides a comprehensive scheme to standardize the evaluation of treatment response after local–regional therapy, thereby guiding management decisions. As per LI-RADS TRA, treated lesions are categorized as viable, nonviable, or equivocal [17] (Fig. 4). An ECA-MRI-based study comprising 36 patients, Chaudhry et al. [18] showed that the LI-RADS TRA performed well in the prediction of both complete (negative predictive value range for residual tumors, 89%–90%) and incomplete (positive predictive value range for residual tumors, 70%–87%) tumor necrosis when equivocal estimates were treated as viable or nonviable, respectively. In addition, the majority of ablated tumors categorized as LR-TR equivocal were confirmed as incompletely necrotic at histopathology, suggesting that sensitivity for incompletely necrotic lesions might be increased if equivocal lesions were regarded as viable. Furthermore, post-treatment nodular, mass-like, or irregular thick tissue in or along the treated lesion with arterial phase hyperenhancement was the most powerful predictor of histopathological necrosis (odds ratio: 142.86).

In summary, several conventional ECA-MRI features depicting either intra- or peri-tumoral alterations may convey prognostic information for HCC patients treated with thermal ablation. As for the application of LI-RADS TRA on ECA-MRI, although potentially promising, further multicenter prospective validation is needed to confirm these clinically meaningful results.

### Gadoxetate disodium-enhanced MRI

#### Conventional imaging markers

Hepatobiliary phase (HBP) images obtained by hepatobiliary-specific contrast-enhanced MRI can visualize the impairment of hepatocyte function and provide vital information for tumor grading before ablation and assessment of tumor activity after treatment. Several studies investigated the prognostic implications of imaging features on pre-ablation EOB-MRI in patients with HCC. For example, in a retrospective, single-center study of 183 patients, Bae et al. [19] reported that satellite nodules on HBP images were independently associated with poor disease-free survival and overall survival, whereas HBP peritumoral hypointensity was predictive of poor overall survival. Another study by Cha et al. [20] assessed the early recurrence patterns in 349 patients after RFA. The authors observed that tumors with arterial rim enhancement plus other targetoid appearances showed significantly higher rates of local tumor progression, intrahepatic distant metastasis and extrahepatic metastasis within 2 years compared with those without arterial rim enhancement. Whereas no differences in outcomes were observed between tumors with arterial rim enhancement only and those without arterial rim enhancement. In addition, Kang et al. retrospectively constructed a risk score for the prediction of local tumor progression after RFA of HCC based on tumor size, tumor margin and HBP peritumoral hypointensity [21].

#### LI-RADS TRA based on EOB-MRI

The first EOB-MRI-based study evaluating the performance of LI-RADS TRA after thermal ablation of small HCC was conducted on a retrospective cohort of 45 patients. The authors reported that LR-TRA after thermal ablation had high interrater reliability (90% agreement, Cohen’s ĸ = 0.75) but unsatisfactory sensitivity (30%) in detecting residual viable tumors [22]. The low accuracy might be attributed to the disruption of local blood flow in the ablated tissue, which could impact the arterial enhancement and washout seen at MRI. These findings emphasized the importance of incorporating histopathology as the gold standard for estimation of ablated HCCs in future studies.

#### Nonhypervascular HBP hypointense nodule

Nonhypervascular HBP hypointense nodule (NHHN) refers to the borderline hepatocellular nodules with the absence of arterial phase hyperenhancement and presence of hypointensity on HBP images (Fig. 4), because the decrease in organic anion transporting polypeptide 8 expression occurs at an earlier step of hepatocarcinogenesis than the typical dynamic vascular alterations of progressed HCC [23]. Previous studies have reported that pre-existing NHHN could develop into hypervascular HCCs during follow-up [24, 25]. The association between the presence of NHHNs and tumor recurrence after RFA of HCC has been explored. Lee et al. [26] retrospectively investigated 139 patients from a single center and reported that the presence of NHHNs was an independent risk factor of HCC recurrence after RFA. Notably, the 5-year cumulative incidences of intrahepatic distant recurrence were significantly higher in patients with NHHNs than in those without, whereas no significant difference was observed in the 5-year cumulative incidences of local tumor progression and extrahepatic metastasis. These findings were in good accordance with other three studies, which demonstrated that the presence of NHHNs was a predictive factor of recurrence [27] or intrahepatic distant recurrence [28, 29]. This could be partly explained by the fact that RFA stimulates distant tumor growth by immunomodulatory processes and proangiogenic pathway [30–32]. Specifically, the “off-target” effect of RFA, which might be secondary to the elevation of cytokines (e.g., interleukin-6 and hepatocyte growth factor) in a response to hepatic regeneration following tissue injury, could possibly promote the acceleration of carcinogenesis in NHHNs [33]. More recently, Lee et al. [34] investigated whether the presence of NHHNs could assist in the decision-making between hepatectomy and RFA in 345 HCC patients. The researchers observed that the presence of NHHNs was a significant predictor of HCC recurrence after both RFA and surgical resection. Moreover, patients without NHHNs achieved better RFS after hepatectomy compared to RFA, whereas patients with NHHNs obtained similar outcomes after the two treatments. These findings highlighted the promising role of MRI in directing the curative treatment selection for early HCCs.

#### Ablation margin

A sufficient ablation margin (AM) surrounding the index tumor is another critical element affecting progression-free survival and overall survival of HCC patients [35–38]. The first study evaluating the utility of post-ablation EOB-MRI in assessing AM was conducted on a retrospective cohort of 95 patients. The authors categorized AM on HBP images into three grades, including AM( +), low-intensity area with continuous high-intensity rim; AM zero, low-intensity area with discontinuous high-intensity rim; and AM( −), low-intensity area extends beyond the high-intensity rim. The cumulative local tumor progression rates in AM( +) HCCs were significantly lower than those in AM zero HCCs [39]. More recently, in a retrospective study of 29 patients, Takeyama et al. [40] reported that the ablation margin status (AM( +), ablation margin completely surrounding the tumor vs. AM zero, a partially discontinuous ablation margin without protrusion of HCC) assessed using fusion images of pre- and post-ablation HBP series was an independent predictor for local tumor progression.

Compared to ECA-MRI, EOB-MRI provide additional information in the prediction and assessment of tumor response after ablation (i.e., HBP images features including the tumor and the peritumoral characteristics). In addition, the ablation margin status assessed by EOB-MRI is a significant predictor of local tumor progression. It is because most HCCs after ablation are not pathologically confirmed, thus EOB-MRI may be an alternative method to determine whether the residual tumor is still viable.

### Contrast enhanced-MRI in combination with contrast enhanced-computed tomography

**Fig. 5:**
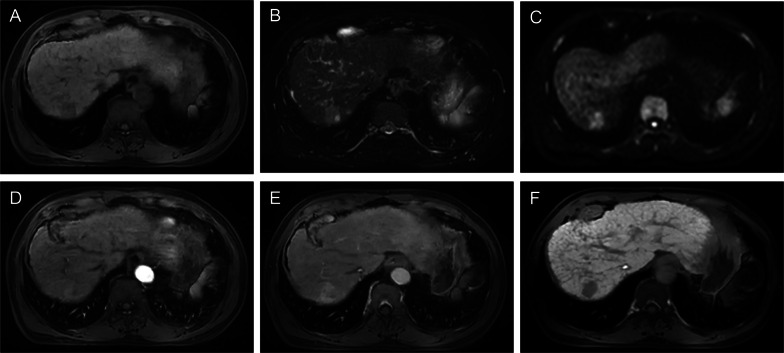
Images in a 62-year-old man with chronic hepatitis B virus infection. **A**–**F** Gadoxetate disodium–enhanced axial MRI scans show a 2.4-cm mass in segment VII (subphrenic location), which shows T1 hypointensity (**A**), mild to moderate T2 hyperintensity (**B**), marked hyperintensity on DWI (b = 800 s/mm^2^) (**C**), mild inhomogeneous enhancement on arterial phase image (**D**), obvious enhancement on portal venous phase (**E**), and demonstrates marked hepatobiliary phase hypointensity (**F**). The recurrence-free survival for this patient was 50 days

Several studies investigated the effectiveness of contrast-enhanced MRI in combination with contrast-enhanced computed tomography (CT) in the estimation of recurrence and survival of HCC patients undergoing ablation. For example, in a recent study of 467 patients, Lee et al. [41] retrospectively evaluated the 10-year overall survival and local tumor progression of RFA for single small (< 3 cm) HCCs. The 5- and 10-year overall survival rates were 83.7% and 74.2%, respectively, and the 5- and 10-year local tumor progression rates were 20.4% and 25.1%, respectively. In addition, local tumor progression was an independent risk factor for overall survival, while periportal and subphrenic locations of HCC and tumor size were independently associated with local tumor progression. Periportal HCC is more prone to recurrence, which is mainly related to the heat-sink effect [42]. This effect prevents a sustained accumulation of heat in the tumor area during the ablation process. In addition, the surgeon may choose a less energetic ablation needle for the procedure to avoid damage to the adjacent vessel wall, which may also lead to inadequate ablation margins [43]. For tumors in specific locations, such as subphrenic HCC (Fig. 5), the increased risk of local tumor progression may be due to the difficulty in placing electrodes along the surface of the liver to obtain sufficient ablation margins for subphrenic tumors [44].In another western cohort of 238 patients, Hermida et al. [16] identified several clinical and radiological factors, including tumor size, multiplicity, steatotic HCC, serum alpha-fetoprotein (AFP) > 100 ng/mL, treatment naivety, Ultrasound (US) guidance, American Society of Anesthesiologists score > 2, for predicting tumor recurrence and/or overall survival after percutaneous thermal ablation. Moreover, a retrospective study by Kawamura et al. [45] demonstrated that enhancement pattern type (heterogeneous enhancement pattern with a septum-like structure and irregularly shaped ring structure enhancement pattern), treatment procedure (touch ablation), and serum AFP ≥ 30 μg/L were independently predictive of intrasubsegmental recurrence. Furthermore, in a prospective study comprising 589 HCC patients, Kondo et al. [46] assessed the prognostic impact of thermal injuries to intrahepatic bile duct after RFA. The authors reported that the bile duct dilatation affecting two or more subsegments after RFA was significantly associated with recurrence and death, suggesting the need for careful evaluation of such posttreatment complication. What’s more, a prospective study comprising 33 patients showed that MRI outperformed multidetector-row CT in the differentiation of ablation margin and index tumor immediately after RFA of HCC. The cumulative incidence of local tumor progression was significantly lower in AM( +) tumors (AM completely surrounding the tumor) on MRI [47]. The above findings suggested that combined enhanced MRI and CT can better characterize the tumor composition and the aggressive biological behavior of HCC, but MRI shows better ability to identify whether the treated lesion with arterial phase hyperenhancement is a treatment response or early tumor recurrence. Despite the clinical relevance, these findings remain to be further validated in multicenter prospective cohorts.

### Diffusion-weighted imaging and diffusion kurtosis imaging

DWI represents a functional MR imaging modality that can characterize water molecule diffusion in tissues by the apparent diffusion coefficient (ADC). It has been recognized that ADC values can reflect the number and proliferation activity of tumor cells [48]. Recent data have shown promising results of DWI in HCC prognostication after ablation. In a study analyzing 136 patients with small HCC, Mori et al. [49] reported that the hypointensity on the apparent diffusion coefficient (ADC) map was independently associated with tumor recurrence and survival after RFA. In addition to this, ADC values also enable quantitative evaluation of RFA efficacy in HCC. For example, Ma et al. [50] retrospectively analyzed 64 patients using ADC histogram analysis and reported that the baseline ADC values could be used as imaging markers for predicting progression-free survival in HCC patients treated with RFA. Another study by Barat et al. [51] showed that a low ADC value at 1 month after RFA was an independent predictor of early local recurrence of HCC, with good predictive accuracy (area under the receiver operating characteristic curve (AUC), 0.860). Moreover, in a retrospective cohort of 105 patients, Hu et al. reported that ADC value and rim enhancement were independently associated with local tumor progression after RFA. By incorporating the above two predictors, a nomogram yielded a concordance index of 0.782 [52]. According to the literature, low ADC values are associated with aggressive tumor behaviors (e.g., poor differentiation and MVI), which may partly explain why HCCs with lower ADC values had poorer clinical outcomes [48, 53]. To better characterize the water diffusion properties in biologic tissues with non-Gaussian form, diffusion kurtosis imaging (DKI) has emerged as a useful technology for assessing the tissue microstructure abnormalities [54]. In a retrospective cohort consisting of 107 HCC patients, Yuan et al. compared the performance between DKI and DWI in prediction of tumor recurrence after RFA. The authors demonstrated that mean kurtosis showed significantly higher accuracy than that of ADC for tumor recurrence prediction (AUC, 0.956 vs. 0.842; *p* < 0.05) [55].

### Fusion imaging

Fusion imaging, which refers to the combination of two different imaging modalities via registration software, has been recently introduced to treatment response assessment for ablative HCC [56]. Compared with traditional visual side-by-side inspection, fusion of pre- and post-RFA images enables more accurate estimation of ablative margin. Recently, Kobe et al. [57] conducted a retrospective study on 43 HCCs to evaluate the ability of fusion of pre-ablation MRI with post-ablation perfusion–CT in assessment of local tumor progression after RFA of HCC. The authors demonstrated that the difference (ablation zone − tumor) of perfusion parameters (normalized peak enhancement, arterial liver perfusion, blood flow, and blood volume) enabled an accurate prediction of local tumor recurrence within 24 h after RFA. Another prospective study by Yoon et al. [58] enrolled 68 patients with 88 HCCs who underwent pre-ablation MRI and post-ablation CT, demonstrating that ablation margin assessment using registration software-assisted inspection was superior to visual evaluation for predicting local tumor progression after RFA. Moreover, Wang et al. [59] explored the ability of EOB-MRI/US fusion imaging in improving the prognosis of HCC patients after RFA, with the ablation area covering two HBP imaging findings (peritumoral hypointensity and irregular protruding margin). The authors showed that HCCs with HBP imaging findings had significantly higher recurrence rates than those without HBP imaging findings. Notably, in HCCs with HBP imaging findings, RFA guided by EOB-MRI/US fusion imaging produced a significantly lower recurrence rate than contrast-enhanced US/US.

### Radiomic analysis

Radiomics is a newly emerging technique of imaging analysis that performs the high-throughput extraction of quantitative features from standard-of-care medical imaging to obtain predictive or prognostic information. Combined with other patient data (e.g., clinical, pathological or genetic characteristics), radiomics displayed potential power to improve prediction accuracy and optimize therapeutic decision-making in various clinical settings [11–13]. Recent research has shown promising results of the radiomics analysis in predicting treatment response and patient outcomes after RFA of HCC. For instance, a pilot study of 34 patients demonstrated that the MRI-based textural features may serve as useful biomarkers for sustained complete response to RFA. In particular, the second-order features (Gray Level Dependence Matrix and Gray Level Co-occurrence Matrix) extracted from equilibrium phase provided the optimal discriminatory performance (AUCs > 0.7) [60]. In a retrospective study of 65 patients, Petukhova-Greenstein et al. [61] reported that multifocality, the appearance of capsular continuity, and a higher radiomic signature based on nodular and perinodular features were associated with poorer PFS in HCC after RFA. Moreover, Wen et al. [62] retrospectively constructed a nomogram for the prediction of early recurrence based on pretreatment platelet count and radiomics signature, with excellent predictive performance (AUC, 0.98). Concurrently, in a retrospective study of 58 HCC patients, Lv et al. [63] developed a model based on tumor shape, ADC value, DWI signal intensity, ΔSI (signal intensity enhancement rate), and radiomics signature for predicting aggressive intrasegmental recurrence after RFA, with good predictive accuracy in both the training (AUC, 0.941) and test (AUC, 0.818) cohorts.

In general, the application of radiomics analysis in predicting treatment response and patient outcomes after RFA of HCC consists mainly of deeper mining of traditional imaging features, and exploration of quantitative MRI features, with the latter being a trend for future research. Despite the great promise, to our knowledge, no radiomic signatures have yet been in widespread clinical use. Current major obstacles for radiomics analysis lie in the standardized data collection and biologic rationales explanation[64]. Future prospective multicenter research is warranted to validate its clinical utility and promote its translation into practice.

## Conclusions and perspectives

There is ample evidence to suggest the potential role of MRI-based qualitative and quantitative parameters in prediction of treatment response and patient prognosis after ablation of HCC, thereby directing a personalized clinical care. Although these initial reports are promising, there are still some shortcomings in the present studies, such as the fact that most studies were single-center or retrospective studies with small sample size. Moreover, there are limited data on MRI-based LI-RADS TRA in the prediction of histologic response and is of limited use in interpreting and validating radiological-pathological correlations. Therefore, future prospective studies in the large multicenter with rigorous designs (e.g., including multiple radiologists with various training and experience levels to assess the interreader reliability and sufficient follow-up period) are needed to look at this issue.

## Data Availability

The figure and table are available from the corresponding author, Prof. Bin Song, upon reasonable request.

## Abbreviations

ADC: Apparent diffusion coefficient
AFP: Alpha-fetoprotein
AM: Ablation margin
AUC: Area under the receiver operating characteristic curve
CT: Computed tomography
DKI: Diffusion kurtosis imaging
DWI: Diffusion-weighted imaging
ECA-MRI: Extracellular contrast agent-enhanced magnetic resonance imaging
EOB-MRI: Gadoxetate disodium-enhanced magnetic resonance imaging
HBP: Hepatobiliary phase
HCC: Hepatocellular carcinoma
LI-RADS: Liver Imaging Reporting and Data System
MRI: Magnetic resonance imaging
MWA: Microwave ablation
NHHN: Nonhypervascular hypointense nodule
RFA: Radiofrequency ablation
TRA: Treatment response algorithm
US: Ultrasound
